# Effects of lifestyle changes including specific dietary intervention and physical activity in the management of patients with chronic hepatitis C – a randomized trial

**DOI:** 10.1186/1475-2891-12-119

**Published:** 2013-08-14

**Authors:** Emilia Rusu, Mariana Jinga, Georgiana Enache, Florin Rusu, Andreea Diana Dragomir, Ioan Ancuta, Ramona Draguţ, Cristina Parpala, Raluca Nan, Irina Sima, Simona Ateia, Victor Stoica, Dan Mircea Cheţa, Gabriela Radulian

**Affiliations:** 1 “Prof. N. Paulescu” National Institute of Diabetes, Nutrition and Metabolic Diseases - Bucharest, Romania; 2 “Carol Davila” University of Medicine and Pharmacy, Bucharest, Romania; 3 Healthy Nutrition Foundation, Bucharest, Romania; 4 “Dr. Carol Davila” Clinical Central Military Emergency Hospital, Bucharest, Romania; 5 Dr. I. Cantacuzino Clinical Hospital, Bucharest, Romania

**Keywords:** Hepatitis C, Diet, Lifestyle change, HOMA-IR, Body mass index

## Abstract

**Background:**

In patients with chronic hepatitis C (CHC), obesity is involved in the pathogenesis of insulin resistance, fatty liver disease and progression of fibrosis. The objective of this study was to compare a normoglucidic low-calorie diet (NGLCD) with a low-fat diet (LFD) among participants with CHC. Aimed to measure the impact of dietary changes in reduction of insulin resistance, obesity but also in steatosis and fibrosis.

**Methods:**

Randomized, controlled trial in three medical centers with assessments at baseline, 6 months and 12 months. Participants were patients over 35 years with chronic hepatitis C (n = 120) with BMI over 25 kg/m^2^. We evaluated the effects of NGLCD *vs*. LFD in weight management and metabolic improvement. The primary endpoint was to measure the impact of dietary changes through nutritional intervention in reversibility of insulin resistance, obesity, steatosis, and fibrosis. We performed anthropometric measurements, fasting glucose profile, serum lipids, liver profile, blood count at baseline, 6 and 12 months. Steatosis was evaluated using ultrasonographic criteria. Liver fibrosis was non-invasively assessed.

**Results:**

After 6 and 12 months of intervention, both groups had a significant decrease in caloric consumption. At 6 months, weight loss was greater in the NGLCD group (−5.02 ± 3.43 kg *vs*. −4.1 ± 2.6 kg; p = 0.002) compared to the LFD group. At 1-year, however, weight loss was similar in both groups (−3.9 ± 3.3 kg *vs*. −3.1 ± 2.6 kg; p = 0.139). At 12 months, fasting plasma glucose, fasting plasma insulin, and HOMA-IR had significant improvements in both groups. With both diets aspartate aminotransferase (AST), alanine aminotransferase (ALT), gamma-glutamyl transpeptidase (GGT) decreased with significant differences; also there were significant improvements in AST/ALT ratio, Forns fibrosis index. The two diets were associated with reduction of both the prevalence and the severity of steatosis (all p < 0.001). At 12 months, total cholesterol, HDL-cholesterol, triglycerides improved in both groups (all p < 0.05).

**Conclusions:**

The present study establishes the benefits of low-calorie diet and low-fat diet in management of patients with hepatitis C regarding improvement of insulin resistance, steatosis and also fibrosis.

Overweight or obese patients with CHC undergoing a lifestyle intervention (specific dietary intervention and physical activity) for 1-year had significant improvements in body weight, lipid and hepatic profile.

**Trial registration:**

PNCI2-3343/41008/2007

## Background

The prevalence of hepatitis C virus (HCV) infection worldwide is estimated at 3% [1]. World Health Organization estimates that the prevalence of HCV in Europe is 1% [1]. In Romania, statistics show that there are 1 million people (4.5% of the population) infected with HCV [2].

Chronic hepatitis C (CHC) can be considered a metabolic liver disease which implies: insulin resistance (IR), increased prevalence of impaired glucose tolerance or type 2 diabetes mellitus (T2DM), changes in lipid metabolism, and a high prevalence of steatosis [3].

As obesity is involved in the pathogenesis of hepatic steatosis and fibrosis progression, one of the important objectives of nutrition management is weight control.

## Methods

### Trial design

This multicenter, randomized controlled trial was conducted from September 2007 to December 2010.

### Participants

Participants were recruited from three hospitals from Bucharest, Romania. The inclusion criteria were: age over 35 years, BMI over 25 kg/m^2^ diagnosis of chronic hepatitis C (CHC infection was defined by the presence of anti-HCV antibodies for a least 6 months and a positive HCV-viremia).

The exclusion criteria were: patients with other etiology of chronic liver disease, hepatitis B, autoimmune liver disease, hemochromatosis, HIV infection, patients with history of hepatotoxic or steatosis-inducing drug use, currently on interferon treatment or during the last 12 months, patients having an alcohol consumption of more than 20 g/day for women and 30 g/day for men, history of pancreatitis.

### Study setting

The study was conducted in Bucharest, the most important commercial urban setting of Romania, with a population of 2 million and an estimated CHC rate of 3.35% in adults (data as of 2007).

### Trial overview

The DIADIPOHEP (Adipocitokynes, link between virus C hepatitis and type 2 diabetes mellitus)) study was approved by the Romanian National Authority for Scientific Research. Written informed consent was obtained from all participants.

Enrollment began in September 2007 and ended in December 2010. Participants were recruited from three hospitals. Eligibility was established through a screening visit that included a physical examination and a review of the patient's medical history. Following completion of baseline assessments, participants were randomized to a normoglucidic low-calorie diet (NGLCD) group, or to a low-fat diet (LFD) group, both with a lifestlye management program.

### Outcome measures

The primary endpoint was to measure the impact of dietary changes in reduction of insulin resistance as well as hepatic steatosis and fibrosis through nutritional intervention. Secondary endpoints included changes in weight, lipid profile (total cholesterol, LDL-cholesterol, HDL-cholesterol and triglycerides), blood pressure (systolic and diastolic), hepatic profile, and renal function (estimated glomerular filtration rate [eGFR]).

### Assessments

We performed anthropometric measurements (weight, height, BMI (body mass index), waist circumference, waist to hip ratio (WHR)) every month.

Body mass index (BMI) was calculated as weight (in kilograms) divided by height (in meters squared). Based on the World Health Organization classification, overweight was defined as BMI between 25 and 29.9 kg/m^2^, and obesity was defined as BMI over 30 kg/m^2^[4]. We also measured waist circumference (in centimeters) at the mid-point of the distance between the 12th rib and iliac crest and hip circumference at the greater trochanters with the legs brought together.

Arterial blood pressure was measured three times at the end of the physical examination with the subject in the sitting position. Participants whose average blood pressure levels were greater or equal to 140/90 mmHg or receiving antihypertensive medication were classified as hypertensive subjects [5].

### Laboratory assays

Fasting blood samples were drawn between 7:00 a.m. and 10:00 a.m.

The biochemical analyses, including fasting serum lipids (total cholesterol (TC), triglyceride (TG), high-density lipoprotein-cholesterol (HDL-C)), glucose profile (fasting plasma glucose (FPG), fasting plasma insulin (FPI), glycated hemoglobin (HbA1c)), liver function tests (aspartate aminotransferase (AST), alanine aminotransferase (ALT), gamma-glutamyl transpeptidase (GGT), alkaline phosphatase, bilirubin, albumin, total protein, International Normalized Ratio (INR)), were performed at baseline, 6 and 12 months with commercially available kits from Roche-Hitachi Systems which were analyzed on a Hitachi 917 autoanalyser. Low-density lipoprotein cholesterol (LDL-C) was calculated using the Friedwald formula (LDL-C = TC − TG/5 + HDL-C) [6].

Serum C-peptide was measured through an electrochemiluminescence immunoassay (Modular Analytics, Roche Diagnostics) with intra- and interassay coefficients of variation of 4.5% and 6.9%, respectively.

Insulin concentration was determined through RIA (Abbott Axsym System, Chicago IL), with intra- and interassay coefficients of variation of 4.5% and 6.9%, respectively.

FPI and C peptide were measured at baseline and 12 months. Insulin resistance (IR) was determined using Homeostasis model assessment (HOMA-IR)(fasting insulin level (mUI/l)x fasting glucose level (mg/dl)/405 [4]; a HOMA-IR index value of more than 2.0 was considered as the criteria of insulin resistance [7].

The oral glucose tolerance test (OGTT) was performed in patients with HbA1c higher than 5.5%. For OGTT, a glucose load equivalent to 75 g anhydrous glucose was given in a total water volume of 250 –300 ml [8]. The glucose drink was consumed over 5 min. Timing for the rest of the test started at the beginning of ingestion. A further blood sample was collected 2 h after the glucose load in order to measure the glucose concentration. Diabetes diagnostic was made according to ADA 2003 criteria [9].

The definition of the metabolic syndrome (MetS) was based on the IDF criteria (central obesity defined as waist circumference over 94 cm in men, over 80 cm in women or BMI over 30 kg/m^2^ plus any two of the following factors: 1. triglycerides (TG) ≥1.695 mmol/l (150 mg/dl) or treatment; 2. high density lipoprotein-cholesterol (HDL-C) lower than 40 mg/dl in men, 50 mg/dl in women or treatment; 3. blood pressure ≥130/85 mmHg or medication; 4. fasting blood glucose ≥ 5.6 mmol/l (100 mg/dl) or medication for diabetes [10].

Liver fibrosis was non-invasively assessed using the Forns fibrosis index (FI) [11]; a value < 4.2 excludes liver fibrosis and a value > 6.9 is a predictor for significant fibrosis. Forns fibrosis index was calculated according to formula: 7.811–3.131 ln[platelet count (10^9^/l)] + 0.781 ln[gamma-glutamyl transpeptidase (GGT) (UI/l)] + 3.467 ln[age (years)] − 0.014[cholesterol (mg/dl)]. The presence of significant fibrosis was predicted with a 96% negative predictive value (NPV) and 66% positive predictive value (PPV) [11].

The AST to platelet ratio index (APRI) was calculated by dividing the AST level (UI/l), expressed as the number of times above the upper limit of normal (ULN), by the platelet count (10^9^/l): AST (/ULN) × 100/platelet count (10^9^/l) [12]. APRI is simpler to use than most of the other indices with similar performance to that of the Fibrotest (FT) and the Forns fibrosis index. This index has been validated in HCV patients [12]. An 86% NPV and an 88% PPV were reported to predict the presence of significant fibrosis and a 98% NPV and a 57% PPV were reported to predict the presence of cirrhosis [12].

Hepatic steatosis (HS) was evaluated using ultrasonographic criteria. Hepatic ultrasound is a sensitive procedure for detecting liver fat (sensitivity 91–100, specificity 93–100) [13]. The severity of echogenicity was graded as follows: grade 0, normal echogenicity; grade 1, slight, diffuse increase in fine echoes in liver parenchyma with normal visualization of diaphragm and intrahepatic vessel borders; grade 2, moderate, diffuse increase in fine echoes with slightly impaired visualization of intrahepatic vessels and diaphragm; grade 3, marked increase in fine echoes with poor or nonvisualization of the intrahepatic vessel borders, diaphragm, and posterior right lobe of the liver.

Estimated glomerular filtration rate (eGFR) was made according to CKD-EPI equation [14]. The CKD-EPI equation, expressed as a single equation, is: GFR = 141 X min(Scr/κ,1)^α^ X max(Scr/κ,1)^-1.209^ X 0.993^Age^ X 1.018 [if female] X 1.159 [if black].

### Randomization

Independently done computer randomization was used to allocate numbers and divide the patients into two groups. Randomization was done by block design to ensure equal numbers in each group for every 4 subjects recruited.

### Diet

All patients received nutrition counseling (NGLCD or LFD) in individual sessions every week in the first 6 months and every month thereafter until 12 months, with biological reevaluation at 6 and 12 months. All patients were required to submit a food journal at the baseline visit (before group allocation), as well as subsequent journals prior to the 6 month, 12 month, and each monthly visit. The food journal covers a 4 day period that includes 2 working days and 2 free days/weekend. Foods were measured using standard measuring cups and spoons and weight for 100 g; the input accuracy of the food journals was confirmed by using food-frequency questionnaires.

No supplements were allowed in this period. Patients who missed more than 30% of dietitian appointments or did not complete the food journals were considered noncompliant and were excluded from final analysis.

Macronutrient intake was calculated using the United States Department of Agriculture's food database (National Nutrient Database for Standard Reference, Release 16–1 and 17, Release dates July 2003 and 2004, Beltsville, MD) [15].

Subjects were required to limit alcohol intake to <20 g/week during the intervention period. Alcohol intake was averaged and recorded as grams per week.

### Normoglucidic low-calorie diet

Dietitian doctors instructed participants to follow a diet comprising approximately 50-60% of daily caloric intake from carbohydrates [16], 25-35% of total calories from fat (less than 7% of total calories from saturated fat, less than 1% trans fatty acids, 10% monounsaturated fatty acids, 5-10% polyunsaturated fatty acids (PUFAs) and less than 300 mg cholesterol per day), proteins 15% of total calories (1.0 to 1.2 g/kg/day) [17], and <5% of caloric intake from simple sugars. Nutrient-rich choices that included whole grains, vegetables and fruit were prioritized. NGLCD was defined as a normoglucidic, normolipidic, normoproteic, low-calorie diet (100–500 kcal less than estimated energy needs).

### Low-fat diet

Restriction of fat intake to 20% of total daily energy uptake with avoidance of trans-fat and saturated fat, up to 20% of the total calories from proteins and 60-65% carbohydrates. Further recommendation included increasing fibre uptake to 30 g per day, and avoiding liquid mono- and disaccharides. Moreover, patients were advised to consume at least 250 to 300 g of fruits, 125 to 150 g of vegetables, and 25 to 50 g of walnuts per day; in addition, they were also encouraged to consume 400 g of whole grains (rice, maize, and wheat) daily and to increase their consumption of olive oil. Compared with normoglucidic low-calorie diet, low-fat diet was defined by a low intake of fat (up to 20% of caloric intake), increased carbohydrate intake up to 60-65% of daily caloric intake, increased fiber intake (30 g/day), and protein intake up to 20%.

### Physical activity

A healthy lifestyle includes regular physical activity (PA). Regular physical activity included 30 minutes of moderate intensity physical activities (e.g. brisk walking, jogging, cycling) for 3–7 days a week, recommended for persons with hepatitis C virus infection without advanced cirrhosis or other metabolic complications [18].

### Energy needs

A high-energy diet is normally recommended for HCV-infected persons [19,20]. Measured energy needs of patients with HCV infection, even in the absence of cirrhosis, are on average higher per unit of lean body mass than the needs of healthy individuals [21]. The following provides two reasonable estimates of energy needs for patients undergoing physiological stress, such as those with infection: 25 to 40 kcal/kg, based on dry weight or an adjusted ideal weight [22] or add 20% to 40% to basal energy expenditure (BEE) using the Harris-Benedict equation [23].

In patients with overweight or obesity, the energy intake was individualized to be 100–500 kcal less than estimated energy needs because we designed it to induce at least a 5-10% weight loss at 6 months and to maintain this weight loss in the subsequent 6 months.

### Statistics

Results for continuous normally distributed data were expressed as mean ± standard deviations (SD). Tests of normality used were Kolmogorov-Smirnov with a Lilliefors significance correction and Shapiro-Wilk statistic. The comparison of mean value at baseline, 6 months and 12 months was performed with paired *t*-test. For continuous nonnormal distribution we used Wilcoxon's rank-sum tests, and data were reported as median ± interquartile ranges. Pearson's *χ*2 tests were used to compare changes in continuous variables from beginning categorical baseline characteristics. Wilcoxon's rank-sum tests were also used to compare changes in continuous variables from baseline to the 12 month. P-value less than 0.05 was considered significant. All statistical analyses were performed using SPSS 19 (copyright IBM).

The primary analysis was intention-to-treat and involved all patients who were randomly assigned [24]. Two patients in the NGLCD group and eight patients in LFD groups were lost to follow up; thus data from 110 patients were available for the intention-to-treat analysis.

## Results

**Figure 1:**
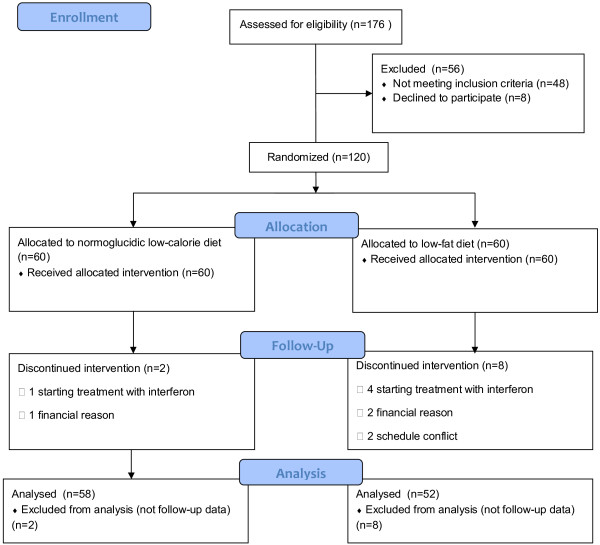
Participant flow diagram.

The flow chart for study participants is presented in Figure 1[25]. The completion rate was 91.6%; 10 of the 120 subjects (8.3%) participating at baseline did not complete the 1-year intervention: 2/60 (3.3%) in the NGLCD group and 8/60 (8.3%) in the LFD group. The dropout rate in the LFD group was significantly greater than that in the NGLCD (p = 0.047). At baseline, in both groups, there were no statistically significant differences between patients who completed the study and those who dropped out.

The baseline characteristics of participants who completed the 1-year intervention are presented in Table 1; participants were similar in sex distribution, smoking, glycemic state, hypertension, dyslipidemia (Table 1). There were no significant differences between the groups (data not shown) for demographic characteristics, marital status, environment, occupation. In both groups, compared with women, men had a higher mean baseline BMI (mean difference 1.96 kg/m^2^ [95% CI 0.13, 3.79]) in NGLCD group and 2.1 kg/m^2^ [95% CI 0.33, 4.06]) in LFD group. All patients were affected by genotype 1.

**Table 1:** Demografic and clinical characteristics at baseline

	**NGLCD (n = 58)**		**LFD (n = 52)**	
Gender	M (n = 26)	F (n = 32)	M (n = 21)	F (n = 31)
Age (years)	52.5 ± 7.1	55.4 ± 9.7	52 ± 8.4	54.9 ± 10.1
BMI (kg/m^2^)	30.6 ± 4.4	29.6 ± 2.3*	30.7 ± 4.2	28.5 ± 2.4**
WC (cm)	95.6 ± 15.4	91.5 ± 12.2	98 ± 12.4	89.02 ± 9.8
Obesity (number, %)	19 (73.1%)	24 (75%)	16 (76.2%)	22 (71%)
Hypertriglyceridemia (number, %)	18 (69.2%)	15 (46.9%)	17 (81%)	17 (54.8%)
HypoHDL-cholesterolemia (number, %)	18 (69.2%)	19 (59.4%)	17 (81%)	15 (48.4%)
Cigarette smoking (number, %)	10 (38.5%)	13 (40.6%)	7 (33.3%)	11 (35.5%)
Diabetes (number, %)	11 (42.3%)	7 (21.9%)	9 (42.9%)	6 (19.4%)
IGT (number, %)	1 (3.8%)	4 (12.5%)	5 (23.8%)	2 (6.5%)
IFG (number, %)	5 (19.2%)	2 (6.3%)	1 (4.8%)	6 (19.4%)
Normal glucose tolerance (number, %)	14 (53.8%)	21 (65.6%)	11 (52.4%)	19 (61.3%)
Hypertension (number, %)	15 (57.7%)	13 (40.6%)	11 (52.4%)	19 (61.3%)
MetS (number, %)	18 (69.9%)	18 (56.3%)	18 (85.7%)	14 (45.2%)***

All p > 0.05 except *, p = 0.036; **, p = 0.022; ***, p = 0.003; Values are expressed as mean ± SD, *NGLCD* normoglucidic low-calorie diet, *LFD* low-fat diet, *BMI* body mass index, *WC* waist circumference, *HDL* high density lipoproteins, *IGT* impaired glucose tolerance, *IFG* impaired fasting glucose, *MetS* metabolic syndrome.

The average age was 54.3 ± 8.6 years in NGLCD group and 54.2 ± 9.3 years in LFD group (p = 0.51).

Overweight was present at baseline in 60.3% (n = 35) patients in NGLCD group and in 55.8% (n = 29) patients in LFD group; the other patients had obesity (p = 0.53 between groups). Overweight was present at 12-months in 55.2% (n = 32) patients in NGLCD group and in 57.7% (n = 30) patients in LFD group; obesity was present in 20.7% (n = 12) patients in NGLCD group and in 23.1% (n = 12) patients in LFD group.

The prevalence of MetS at baseline was similar between groups (61.5% in NGLCD (n = 36) *vs*. 61.5% in LFD (n = 32), p = 0.55) but higher in men from LFD group than in women from the same group (85.7% (n = 18) *vs*. 45.2% (n = 14), p = 0.003).

### Kilocalories and macronutrients

At baseline, analysis of the 4-day food journal showed that calorie intake of the two groups was not significantly different (2247 ± 160 Kcal/day *vs*. 2213 ± 157 Kcal/day in the NGLCD and LFD, p = 0.261). The results were similar for the baseline protein (16.2 ± 1.6% *vs*. 16.3 ± 1.6%, p = 0.506), lipid (33.9 ± 3.52% *vs*. 33.2 ± 3.51%, p = 0.1) and carbohydrate intake (49.8 ± 3.4% *vs*. 50.2 ± 3.3%, p = 0.8) in the NGLCD and LFD group, respectively.

Significant reduction in carbohydrate, protein and lipid intake was observed in the NGLCD group both at 6 months and 12 months (all p < 0.001) (Table 2). In LFD group we observed a significant reduction of calories and lipid intake and an increase of protein and carbohydrates intake during the intervention period (all p < 0.001 at 12 months) (Table 2).

**Table 2:** **Kilocalories and macronutrients**, **comparison at baseline and after 6 and 12 months**

	**NGLCD**					**LFD**				
	**Baseline**	**6 months**	**p0**-**6**	**12 months**	**p0**-**12**	**Baseline**	**6 months**	**p0**-**6**	**12 months**	**p0**-**12**
**Kcalories**	2247.9 ± 160.3	1909.8 ± 251.3	0.001	1842.8 ± 255.1	0.0001	22213.6 ± 157.7	1900.5 ± 226.7	0.0001	1845.2 ± 240.8	0.001
**Protein (g)**	91.3 ± 10.1	70.6 ± 11	0.0001	70.7 ± 11.8	0.0001	91 ± 10	90.9 ± 18.1	0.954	90.6 ± 16.8	0.861
**Protein (%)**	16.2 ± 1.6	14.8 ± 1.3	0.001	15.3 ± 1	0.0001	16.4 ± 1.6	19.9 ± 1.5	0.0001	20 ± 1.3	0.0001
**Carbohydrate (g)**	280.2 ± 30	265 ± 38.7	0.001	251.6 ± 40.4	0.0001	278.7 ± 32.2	285.2 ± 38.5	0.293	274.8 ± 36.6	0.513
**Carbohydrate (%)**	49.8 ± 3.4	55.3 ± 2.2	0.001	54.4 ± 2.5	0.0001	50.2 ± 3.3	60.1 ± 1.6	0.032	59.7 ± 1.3	0.0001
**Lipid (g)**	84.6 ± 10.3	63.5 ± 9.4	0.001	61.4 ± 8.1	0.0001	81.6 ± 1.2	44.6 ± 8.8	0.0001	43.3 ± 7.04	0.0001
**Lipid (%)**	33.9 ± 3.5	29.9 ± 2.4	0.0001	30.1 ± 2.9	0.0001	33.2 ± 3.5	19.6 ± 1.8	0.0001	20.2 ± 1	0.0001

Values are expressed as mean ± SD.

*NGLCD* normoglucidic low-calorie diet, *LFD* low-fat diet.

After 6 and 12 months of intervention, both groups had a significant decrease in caloric consumption (Table 2), without differences between groups (p = 0.839 at 6 months, and p = 0.96 at 12 months); at 12 months, fat consumption in LFD patients was significantly lower (43.3 ± 7.04 g/day *vs*. 61.4 ± 8.1 g/day).

### Weight loss

At 6 months, weight loss was greater in the NGLCD group (−5.02 [95% CI −5.9, -4.1]kg *vs*. −4.1 [95% CI −4.8, -3.3]kg; p = 0.002) compared to the LFD group. At 1-year, however, weight loss was similar in both groups (−3.9 [95% CI −4.8, -3.1]kg *vs*. −3.1 [95% CI −3.8, -2.3]kg; p = 0.139). Most of the weight loss occurred in the first 6 months. After 12 months, patients slowly regained weight after no longer being under observation on a regular basis. We also found no significant sex differences for changes in weight. A total of 18 patients in LFD group (34.6%) and 21 patients in NGLCD group (36.2%) continued to lose weight from 6 months to 1 year. At 1 year, 29.3% (n = 17) patients in NGLCD group and 30.8% (n = 16) patients in LFD group had lost between 5-10% of their initial body weight; 10.3% (n = 6) patients in NGLCD group and 1.9% (n = 1) patients in LFD group lost over 10% of their initial weight (p = 0.33).

There were no significant differences between the groups in body weight changes, BMI, waist circumferences at 12 months (Table 3).

**Table 3:** Metabolic syndrome parameters

	**NGLCD**			**LFD**		
	**Baseline**	**12 months**	**Mean change**	**Baseline**	**12 months**	**Mean change**
WC (cm)	92.7 ± 13.6	89.5 ± 11.9	−3.3 ± 3.6 [−4.3, -2.3]	92.4 ± 11.7	89 ± 10.6	−3.4 ± 3.1 [−6.7, -0.96]
BMI (kg/m^2^)	29.4 ± 3.5	28.9 ± 3.6	−1.4 ± 1.1 [−1.7, -1.1]	29.4 ± 3.4	28.3 ± 3.4	−1.1 ± 0.9 [−1.3, -0.8]
Weight (kg)	84.07 ± 13.2	80.09 ± 13.8	−3.9 ± 3.3 [−4.8, -3.1]	83 ± 11.1	79.8 ± 11.5	−3.1 ± 2.6 [−3.8, -2.3]
Triglycerides (mg/dl)	157.4 ± 50.8	128.5 ± 31	−28.9 ± 5.09 [−39.1, -18.8]	164.6 ± 47.8	134.1 ± 32.3	−30.4 ± 38.7 [−41.2, -19.7]
HDL-cholesterol (mg/dl)	42.7 ± 11.3	46 ± 10.2	3.3 ± 3.8 [4.3-2.3]	42.3 ± 10.3	46.7 ± 8.9	4.4 ± 4.03 [3.3, 5.5]
FPG (mg/dl)	104.1 ± 27.9	96.9 ± 19.7	−7.2 ± 11.2 [−10.2, -4.2]	103.7 ± 29.2	96.5 ± 19.1	−7.2 ± 13.1 [−10.8, -3.5]
Cholesterol (mg/dl)	212.4 ± 40.4	190.2 ±20.2	−22.1 ± 28.6 [−29.5, -14.7]	209.1 ± 37	185 ± 16	−24.1 ± 27.5 [−31.8, -16.4]
SBP (mmHg)	130.8 ± 11.2	125.9 ± 12.4	−4.8 ± 8.7 [−6.9,-2.7]	143.7 ± 12.2	129.4 ± 11.2	−4.9 ± 6.1 [−6.5, -3.3]
DBP (mmHg)	78.8 ±8.04	76.1 ± 8.6	−2.6 ± 10.6 [−5.2,-0.06]	84.8 ± 9.7	76.7 ± 10.3	−3.8 ± 11.1 [−6.7, -0.96]

Values are means ± SD; for mean change the results were expressed as means ± SD and 95% confidence intervals.

*NGLCD* normoglucidic low-calorie diet, *LFD* low-fat diet, *WC* waist circumference, *BMI* body mass index, *HDL* high density lipoproteins, *FPG* fasting plasma glucose, *SBP* systolic blood pressure, *DBP* diastolic blood pressure.

All p < 0.005 for within group change from baseline to 12 months; All p >0.05 for difference between groups at baseline and 12 months.

### Physical activity

Before the start of the study all patients were sedentary, not involved in any form of regular exercise (in NGLCD, PA was 28.7 ± 18.7 min/week, in LFD, PA was 29.5 ± 19.3 min/week).

The degree of PA increased in the first 6 months in both groups statistically significant; even if in the next 6 months PA decreased, the difference between baseline and 12-months remained significant in both groups (for NGLCD the difference was 56.5 min/week [95% CI, 46.4, 66.6] and 39 min/week [95% CI 28.7, 49.3] for LFD.

Comparing the two groups, significant differences were found only at the 12-months visit, when patients in the NGLCD group continued to be more active (16.7 min/week [95% CI 1.8, 31.5]).

### Effect of diet programs on insulin resistance

In NGLCD group, after the 12-month intervention we have seen reductions of 5.5% (95% CI 3.4, 7.6) in FPG and of 27.4% (95% CI 20.3, 34.5) in FPI; in LFD group we also observed a reduction of 5.2% (CI95% 2.9, 7.4) for FPG and of 21.6% (95% CI 15.3, 28) for FPI, respectively. Pairwise analyses test showed significant differences in changes of FPG and FPI for the two groups (Table 4).

**Table 4:** Glucose metabolism parameters

	**NGLCD**			**LFD**		
	**Baseline**	**12 months**	**Mean change**	**Baseline**	**12 months**	**Mean change**
FPG (mg/dl)	104.1 ± 27.9	96.9 ± 19.7	−7.2 ± 11.2 [−10.2, - 4.2]	103.7 ± 29.2	96.5 ± 19.1	−7.2 ± 13.1* [−10.8, -3.5]
FPI (μU/ml)	14.2 (11.5-21.8)	9.1 (6.2-16.9)	−7.6 ± 11.5* [−10.7, -4.6]	12.8 (8.7-18.8)	9.15 (6.06-17.08)	−5.2 ± 9.2* [−7.8, -2.6]
HOMA-IR	3.33 (2.3-5.9)	1.99 (1.45-4.04)	−2.4 ± 4.1 * [−3.5, -1.3]	3.06 (1.93-5.11)	2.04 (1.3-4.4)	−1.8 ± 3.9* [−2.9, -0.7]
C Peptide (ng/ml)	2.31 (2.01-2.8)	2.34 (2.08-2.9)	−0.03-0.2 ** [−0.09, -0.03]	2.4 (2.07-2.97)	2.41 (2.1-3.1)	−0.002 ± 0.2*** [−0.004, 0.01]
HbA1c (%)	5.7 ± 0.7	5.6 ± 0.7	−0.11 ± 0.3 * [−0.02, -0.2]	5.6 ± 0.5	5.5 ± 0.5	−0.09 ± 0.2* [−0.06, 0.05]

Values are means ± SD; for variables abnormally distributed we used median ± interquartile ranges (25th and 75th percentiles).

All p >0.05 for difference between groups at baseline and 12 months; *, p < 0.05 for mean change baseline, 12 months; **, p = 0.295; ***, p = 0.925.

*NGLCD* normoglucidic low-calorie diet, *LFD* low-fat diet, *FPG* fasting plasma glucose, *FPI* fasting plasma insulin, *HOMA-IR* homeostasis model assessment of insulin resistance, *HbA1c* glycosylated hemoglobin.

Insulin resistance determined through HOMA-IR improved by 31.5% (95% CI 24.6, 38.5) in the NGLCD group; HOMA-IR improved also in the LFD group (25.7% (CI95% 19.3, 32.1) (p = 0.219 between groups).

There were no significant differences in the 12-month percentage changes in FPG, FPI, C peptide, homeostasis model assessment for insulin resistance and homeostasis model assessment for β-cell function, between groups. After adjustment for body weight lost, HOMA-IR showed an improvement with both diets (p = 0.026, respectively p = 0.03).

### Effects of diet programs on hepatic profile

The liver function tests at baseline and after 12 months for NGLCD and LFD groups are presented in Table 5.

**Table 5:** Parameters for hepatic function

	**NGLCD**			**LFD**		
	**Baseline**	**12 months**	**Mean change**	**Baseline**	**12 months**	**Mean change**
AST (IU/l)	48 (22–65.5)	40.5 (24.6-60.7)	−6.2 ± 18.2* [−11,1,-1.4]	58 (29.5-85.7)	47.5 (29–62)	−8.7 ± 21.6^#^ [−14.8, -2.7]
ALT (IU/l)	57.5 (22–78)	42 (27.3-68)	−10.6 ± 19.8* [−15.9, -5.4]	67 (41.2-102)	46 (33–67.7)	−11.8 ± 32.2^#^ [−20.8, -2.8]
GGT (IU/l)	46.5 (29–67.5)	39.5 (23.7-55)	−9 ± 16 * [−13.2, -4.8]	62 (45–76)	42 (35–61.5)	−12.1 ± 12.4^#^ [−15.6, -8.6]
Albumin (g/dl)	4.3 ± 0.3	4.3 ± 0.3	0.06 ± 0.3 ** [−0.01,-0.14]	4.2 ± 0.4	4.2 ± 0.3	0.06 ± 0.3^##^ [−0.02, 0.15]
AST/ALT	0.95 ± 0.37	0.96 ± 0.26	0.01-0.3 *** [−0.07,-0.09]	0.89 ± 0.31	0.92 ± 0.2	0.02 ± 0.32^#^ [−.06, 0.12]
APRI	0.47 (0.3-0.82)	0.45 (0.27-0.69)	−0.1 ± 0.27* [−0.17, -0.03]	0.73 (0.31-0.93)	0.53 (0.28-0.71)	−0.13 ± 0.32^###^ [−0.2, -0.1]
FI	5.5 ± 1.1	5.3 ± 1.1	−0.2 ± 0.35* [−0.29, -0.1]	5.5 ± 1.3	5.3 ± 1.3	−0.2 ± 0.31^#^ [−0.3, -0.1]

Values are means ± SD; for variables abnormally distributed we used median ± interquartile ranges (25th and 75th percentiles).

All p >0.05 for difference between groups at baseline and 12 months; *, p < 0.05 for mean change baseline, 12 months; ^#^, p < 0.05 for mean change baseline, 12 months; **, p = 0.295; ***, p = 0.925; ^##^, p = 0.539; ^###^, p = 0.108.

*NGLCD* normoglucidic low-calorie diet, *LFD* low-fat diet, *AST* aspartate aminotransferase, *ALT* alanine aminotransferase, *GGT* gamma-glutamyl transpeptidase, *APRI* AST to platelet ratio index, *FI* Forns fibrosis index.

With both diets AST, ALT, GGT decreased with significant differences; also AST/ALT ratio, APRI score and Forns index had significant improvements. Albumin and bilirubin levels were not significantly changed (Table 5).

In order to assess the effects of weight loss on liver function parameters patients were stratified according to degree of weight loss (weight gain, 1-5% weight loss, 5-10% weight loss, and more than 10% weight loss). Both diets proved to be efficacious in the improvement of liver function parameters.

### In NGLCD patients group

In patients with less than 5% loss of baseline weight (n = 30) (−2.1 kg [95% CI −2.5, -1.75]) there was an improvement of ALT levels (−15.1 [95% CI −22.8, - 7.5]), GGT levels (−6.4 [95% CI [−10.9, -1.9]), AST/ALT ratio (−0.07 [95% CI, -0.002, -0.14]) and Forns index (−0.17 [(95% CI, -0.005, -0.28]) (Table 6).

**Table 6:** Groups of weight loss and liver function parameters in patients with NGLCD

		**Weight gain**		**1-5% weight loss**		**5-10% weight loss**		**Weight loss >10%**		**p**
		**Mean**	**SD**	**Mean**	**SD**	**Mean**	**SD**	**Mean**	**SD**	
PA (min/week)	Baseline	17.0	3.9	27.5	16.2	34.4	23.6	28.7	20.8	0.310
	12-months	46.0	21.0	74.1	39.7	106.2	40.7	114.5	26.6	0.002
Weight (kg)	Baseline	85.4	15.0	85.8	15.1	82.9	11.1	77.9	6.7	0.592
	12-months	86.5	14.5	83.6	14.7	76.4	10.7	67.7	6.2	0.024
BMI (kg/m^2^)	Baseline	28.7	3.8	29.6	3.1	30.4	4.6	27.2	0.8	0.295
	12-months	29.1	3.6	28.9	3.0	28.0	4.5	23.6	0.6	0.011
AST (IU/l)	Baseline	44.8	25.0	51.8	31.7	61.8	45.2	32.3	16.9	0.336
	12-months	35.1	17.9	46.3	22.4	51.9	36.4	35.7	12.5	0.460
ALT (IU/l)	Baseline	41.5	24.9	68.7	42.0	61.3	44.9	35.2	26.7	0.213
	12-months	31.5	14.5	53.6	28.0	54.9	31.4	34.2	19.2	0.168
Albumin (g/dl)	Baseline	4.4	0.5	4.3	0.3	4.2	0.4	4.4	0.3	0.430
	12-months	4.3	0.3	4.4	0.4	4.4	0.4	4.5	0.2	0.744
Bi (g/dl)	Baseline	0.6	0.3	0.7	0.2	0.6	0.2	0.6	0.1	0.566
	12-months	0.6	0.3	0.6	0.2	0.6	0.2	0.6	0.1	0.653
GGT (IU/l)	Baseline	36.4	23.7	58.4	31.1	59.4	47.8	39.3	26.1	0.403
	12-months	30.8	19.2	51.9	34.2	46.9	29.3	24.3	8.0	0.152
AST/ALT	Baseline	1.2	0.4	0.8	0.3	1.1	0.5	1.1	0.5	0.050
	12-months	1.1	0.2	0.9	0.2	1.0	0.3	1.2	0.5	0.075
FI	Baseline	5.5	0.7	5.8	1.2	5.6	1.2	4.6	0.5	0.160
	12-months	5.3	0.5	5.6	1.1	5.4	1.3	4.3	0.5	0.086
APRI	Baseline	0.6	0.4	0.7	0.5	0.9	0.8	0.4	0.2	0.419
	12-months	0.5	0.3	0.6	0.4	0.7	0.6	0.4	0.2	0.596

*NGLCD* normoglucidic low-calorie diet, *PA* physical activity, *BMI* body mass index, *AST* aspartate aminotransferase, *ALT* alanine aminotransferase, *Bi* bilirubin, *GGT* gamma-glutamyl transpeptidase, *AST/ALT* aspartate aminotransferase/alanine aminotransferase ratio, *FI* Forns fibrosis index, *APRI* AST to platelet ratio index.

In patients who lost between 5-10% of baseline weight (n =17, 29.3%) there was an improved GGT, alkaline phosphatase, Forns index, and APRI (Table 6).

In patients from NGLCD group who have lost more than 10% of the body weight (n = 6) there was a significant improvement of non-invasive index of liver fibrosis (Forns index) (Table 6).

### In LFD patients group

In patients with weight gain (n = 4, 7.7%) we observed a slight improvement of Forns index, probably obtained in the context of reduced fat consumption (Table 7).

**Table 7:** Groups of weight loss and liver function parameters in patients with LFD

		**Weight gain**		**1-5% weight loss**		**5-10% weight loss**		**Weight loss >10%**		**p**
		**Mean**	**SD**	**Mean**	**SD**	**Mean**	**SD**	**Mean**	**SD**	
PA (min/week)	Baseline	19.5	7.1	30.5	19.0	31.2	22.2	15.0	.	0.620
	12-months	33.8	11.1	71.5	36.6	70.6	34.5	85.0	.	0.229
Weight (kg)	Baseline	88.9	15.4	82.8	11.0	81.2	10.5	96.0	.	0.412
	12-months	90.3	15.2	80.8	11.1	75.2	10.3	84.0	.	0.090
BMI (kg/m^2^)	Baseline	30.4	4.1	29.2	3.2	29.5	3.9	32.4	.	0.774
	12-months	30.9	4.1	28.5	3.2	27.4	3.8	28.4	.	0.334
AST (IU/l)	Baseline	61.5	56.2	60.0	35.3	65.1	33.0	48.0	.	0.950
	12-months	63.7	57.8	50.3	31.5	55.7	31.0	36.0	.	0.811
ALT (IU/l)	Baseline	63.3	43.4	74.0	44.6	73.2	39.5	69.0	.	0.972
	12-months	77.1	72.9	56.0	40.3	67.8	41.0	42.0	.	0.671
Albumin (g/dl)	Baseline	4.3	0.5	4.2	0.5	4.3	0.4	3.8	.	0.692
	12-months	4.4	0.3	4.3	0.3	4.4	0.3	3.7	.	0.186
Bi (g/dl)	Baseline	0.7	0.2	0.7	0.3	0.6	0.2	0.8	.	0.579
	12-months	0.7	0.1	0.7	0.2	0.6	0.2	0.6	.	0.514
GGT (IU/l)	Baseline	71.3	55.5	59.9	28.0	73.1	36.3	55.0	.	0.592
	12-months	67.5	67.5	48.5	26.6	57.3	34.5	46.0	.	0.654
AST/ALT	Baseline	0.9	0.2	0.9	0.3	1.0	0.4	0.7	.	0.763
	12-months	0.9	0.2	1.0	0.2	0.9	0.2	0.9	.	0.424
FI	Baseline	5.4	1.7	5.6	1.4	5.7	1.3	4.6	.	0.863
	12-months	5.2	1.8	5.3	1.4	5.5	1.3	4.6	.	0.904
APRI	Baseline	0.7	0.6	0.8	0.6	0.8	0.5	0.3	.	0.866
	12-months	0.7	0.6	0.6	0.6	0.7	0.4	0.3	.	0.887

*LFD* low fat diet, *PA* physical activity, *BMI* body mass index, *AST* aspartate aminotransferase, *ALT* alanine aminotransferase, *Bi* bilirubin, *GGT* gamma-glutamyl transpeptidase, *AST/ALT* aspartate aminotransferase/alanine aminotransferase ratio, *FI* Forns fibrosis index, *APRI* AST to platelet ratio index.

In those with less than 5% loss of baseline weight (n = 31, 59.6%) we found improvements in AST, ALT, total bilirubin, INR, Forns index, APRI (Table 7).

In patients with 5-10% loss of baseline weight (n = 16, 30.8%) we found significant improvements in GGT and Forns index, with an improvement in the remaining parameters, but it was not statistically significant (Table 7).

### Steatosis

Fatty liver disease is common in patients with CHC. In our study 52.7% patients (n = 58) presented hepatic steatosis, 60.3% (n = 35) patients in NGLCD group and 46.2% (n = 24) patients in LFD group (p = 0.097).

The two diets were associated with reduction of both prevalence and severity of steatosis (all p < 0.001) without significant differences between groups; in NGLCD group - mild: 68.6% *vs*. 77.4%, moderate: 25.7% *vs*. 22.6%, severe: 5.7% *vs*. 0%; in LFD group - mild: 58.3% *vs*. 76.2%, moderate: 29.2% *vs*. 23.8%, severe: 12.5% *vs*. 0%.

This reduction in prevalence and severity of liver steatosis resulted in a significant diminution of serum triglycerides in NGLCD and of serum ALT levels in LFD group (p = 0.045, respectively p = 0.03). Such regression of steatosis occurred even in absence of weight normalization.

### Effects of diet programs on metabolic syndrome parameters and fasting lipid profiles

At baseline, metabolic syndrome (≥3 criteria) was present in 62.1% (n = 36) patients in NGLCD and in 61.5% (n = 32) patients in LFD (p = 0.55). At 12 months, all parameters associated with the metabolic syndrome improved in both groups (all p < 0.005). At 12-months only 25.9% (n = 15) form patients receiving NGLCD and 26.9% (n = 14) form patients receiving LFD showed MetS; we did not find differences between groups at baseline and 12 months (Table 3).

### Effects of diet programs on renal profile

There were no differences between the two diets regarding the changes in renal function (eGFR, creatinin, urea). In both groups, there were no associations between the changes in protein intake (g/day) and the change in eGFR (*r* = 0.04, *p* = 0.29) or creatinin (*r* = 0.07, *p* = 0.34).

## Discussion

This RCT demonstrated the benefits of both normoglucidic low-calorie and low-fat diets in individuals with CHC. Our results indicated that after 1 year, overweight and obese patients with CHC had similar weight reduction with both diets. The dropout rate in LFD was significantly greater than that in NGLCD. Similar to prior studies, we observed a faster weight loss after initiation of a NGLCD and equivalent weight loss after 1 year [26].

This study demonstrated that lifestyle changes (NGLCD or LFD and physical activity) improved the anthropometric parameters, glucose parameters and lipid and liver profiles. Further improvement was noted in the results of non-invasive liver fibrosis testing, as well as improvement of the prevalence and severity of hepatic steatosis.

The prevalence of MetS in our study was higher than previously published in Romania [27] and in Europe, most likely because we included overweight patients (BMI over 25 kg/m2). At baseline 61.5% of patients belonging to the NGLCD group and 61.5% to the LFD group presented MetS. In the largest retrospective survey (239 HCV-positive subjects) 16.7% had metabolic syndrome [28]. In other studies [29,30], the prevalence of metabolic syndrome in chronic HCV-infected patients ranged from 4.1 to 44% [31].

Even the weight loss at 12 months wasn’t spectacular (−3.9 [95% CI −4.8, -3.1]kg in NGLCD *vs*. −3.1 [95% CI −3.8, -2.3]kg in LFD) there was a reduction in MetS prevalence (25.9% in NGLCD group and 26.9% in LFD group). In CHC patients lifestyle changes through medical nutritional therapy and physical activity led to an improvement in all metabolic parameters: reduced insulin resistance, lower blood glucose, lower triglycerides, total serum cholesterol, LDL-C, increased HDL-C, reducing systolic and diastolic blood pressure.

Modest weight loss of 5–10% body weight is known to reduce insulin resistance in obese individuals [32].

In our study a normoglucidic low-calorie diet (with limited refined carbohydrates and sugar intake, and increased fruits, vegetables and whole grains intake) was accompanied by improvement in insulin resistance (HOMA-IR) lipid and liver profile.

The metabolic changes induced by the low-fat, high carbohydrate, high protein diet were associated with similar weight losses, improved lipid and glucose profiles, however there were no adverse changes in renal function parameters but the compliance to this diet was lower (drop out rate was almost double).

Thus, even if macronutrient intake was different, there were similar improvements in glycemia and insulin resistance, indicating that in the context of tolerable diets and weight loss, mild variations in nutrient fuels have limited impact on glucose metabolism.

In overweight/obese patients with steatosis who subsequently lost weight, liver-related abnormalities improved [33]. Although weight loss may be difficult to achieve and sustain, the patients who did manage to lose weight showed a reduction in steatosis and abnormal liver enzymes as well as improvement in liver fibrosis, despite the persistence of the virus [34]. Lifestyle changes are deemed to be additive to proper antiviral treatment schedules, which remain the standard of care [31].

The effects of lifestyle changes on hepatic inflammation and fibrosis varied [35,36], only one study showed significant improvement [36].

In patients with steatosis, lifestyle changes (diet and exercise) were associated with improvement of ALT levels [35] and steatosis [35,36].

Recently a semiquantitative index used to assess steatosis was validated against histology [37] and proved useful in the specific setting of lifestyle interventions [38].

Limitations of the study are: we used non-invasive methods to estimate steatosis and fibrosis in patients with CHC, and these indices are less sensitive and specific in these patients; the analysis and presentation of only detailed food journals may bias the estimate of food intake; recruited patients were overweight (BMI > 25 kg/m^2^) thus the prevalence of MetS was higher.

At this point only lifestyle interventions can be recommended to improve metabolic syndrome and obesity associated with chronic hepatitis C, but their effect on treatment response and long term outcome requires further study.

Moderate exercise is recommended for all persons with hepatitis C who did not experience advanced cirrhosis or other metabolic complications [17,39,40]. In the present study, changes in food intake and the increase of physical activity were sustainable, associated with long-term metabolic benefits. In some studies, patients with CHC who participated in light or moderate exercise programs reported an improvement in some symptoms such as nausea, fatigue, depression and appetite [17,35,36].

An important issue related to long-term of dietary interventions is that adherence decreases over time and therefore achieving the treatment goals involves an individualized education program, structured and continuously adapted to the socio-biological and family environment, with patient’s involvement in his own treatment.

Long-term benefits can be confirmed only by large studies over a longer period of time, where the patient has adopted the habit of an optimal lifestyle.

## Conclusions

The present study establishes the benefits of the low-calorie diet and low-fat diet in management of patients with hepatitis C regarding improvement of insulin resistance, steatosis and also liver fibrosis.

Overweight or obese patients with hepatitis C undergoing a lifestyle intervention (specific dietary intervention and physical activity) for 1-year had significant improvements in body weight, lipid and hepatic profiles.

## Abbreviations

ALT: Alanine aminotransferase; APRI: AST to platelet ratio index; AST: Aspartate aminotransferase; BMI: Body mass index; DBP: Diastolic blood pressure; FI: Forns fibrosis index; FPG: Fasting plasma glucose; FPI: Fasting plasma insulin; GGT: Gamma-glutamyl transpeptidase; HbA1c: Glycosylated hemoglobin; HDL: High density lipoproteins; HOMA-IR: Homeostasis model assessment of insulin resistance; IFG: Impaired fasting glucose; IGT: Impaired glucose tolerance; LFD: Low-fat diet; MetS: Metabolic syndrome; NGLCD: Normoglucidic low-calorie diet; SBP: Systolic blood pressure; WC: Waist circumference; NGLCD: Normoglucidic low-calorie diet.

## Competing interest

The authors declare that they have no competing interest.

## Authors’ contributions

Conception and design: ER; Providing study materials and inclusion of patients: ER, GR, FR, GE, ADD, MJ, IA, RD, CP, RN, IS, SA, VS, DMC, GR; Data collection and assembly: GE, FR, ER, ADD; Data analysis and interpretation: ER, DMC, MJ, GR; Manuscript elaboration: ER; Final approval of the manuscript: ER, MJ, GE, FR, ADD, IA, NC, PA, RN, IS, SA, VS, DMC, GR. All authors read and approved in the final manuscript.
